# Oxidative Stress in Patients before and after On-Pump and Off-Pump Coronary Artery Bypass Grafting: Relationship with Syntax Score

**DOI:** 10.1155/2021/3315951

**Published:** 2021-07-30

**Authors:** Petar Vukicevic, Aleksandra Klisic, Vojislava Neskovic, Luka Babic, Aleksandar Mikic, Natasa Bogavac-Stanojevic, Milos Matkovic, Vladimir Milićević, Nemanja Aleksic, Jelena Kotur-Stevuljevic

**Affiliations:** ^1^Clinic for Cardiac Surgery, Military Medical Academy, Belgrade, Serbia; ^2^University of Defense, Medical Faculty of the Military Medical Academy, Belgrade, Serbia; ^3^Primary Health Care Center, University of Montenegro-Faculty of Medicine, Podgorica, Montenegro; ^4^Clinic for Anesthesiology and Critical Care, Military Medical Academy, Belgrade, Serbia; ^5^Clinic for Cardiac Surgery, UC Clinical Centre, Belgrade, Serbia; ^6^University of Belgrade-Faculty of Medicine, Belgrade, Serbia; ^7^Department for Medical Biochemistry, University of Belgrade-Faculty of Pharmacy, Belgrade, Serbia; ^8^Department for Cardiac Surgery, University Clinical Center of Serbia, Belgrade, Serbia

## Abstract

**Objective:**

Coronary artery bypass grafting (CABG) represents the significant source of increased oxidative stress (OS). We aimed to follow the OS status parameters (i.e., ischemia-modified albumin (IMA), malondialdehyde (MDA), superoxide anion, prooxidant-antioxidant balance (PAB), total oxidant status (TOS), total antioxidant status (TAS), and superoxide-dismutase (SOD)) change through the predefined study times in two different surgical procedures, i.e., cardiopulmonary bypass (CPB) and off-pump coronary artery bypass grafting (OPCAB). Additionally, we aimed to investigate those OS status parameters in specific study times according to SYNTAX score (SS), an established angiographic score for evaluating the extensity and severity of coronary artery disease. *Patients and Methods*. A total of 107 patients that were planned to undergo CABG were included (i.e., 47 patients in OPCAB and 60 patients in CPB group). Blood samples were taken at 6 time intervals: before surgery (t1), immediately after intervention (t2), 6 h (t3), 24 h (t4), 48 h (t5), and 96 h after termination of the operation (t6).

**Results:**

IMA levels were higher in CPB than that in OPCAB baseline and rose in CPB group in t2 point. TOS decreased in both study groups, compared to baseline values, but without statistical significance. Superoxide anion and PAB significantly increased in t3-t6 study times, in both groups. MDA significantly increased only in CPB group in t5 and t6 interval. MDA was significantly higher in CPB group compared to OPCAB in t6 study point. CPB patients had significantly lower TAS compared to OPCAB patients at the beginning and in t2 and t3 study points. They also had significantly lower SOD activities compared to OPCAB, baseline, and in several study points. Moreover, TAS, SOD, and TAS/TOS ratio were significantly lower, whereas PAB and TOS/TAS were significantly higher in patients with high SS compared to corresponding groups. SOD activity, IMA, and TAS level were the best predictors of high SS.

**Conclusion:**

CPB patients were in more severe ischemia baseline than OPCAB group and IMA rose in CPB patients immediately after the surgery end, but not later. Also, the antioxidant status was significantly lower, whereas the prooxidant status was significantly higher in patients with high SS compared to corresponding groups. SOD activity, IMA, and TAS level were the best predictors of CAD (as determined with SS), showing that SOD and IMA had very good discriminatory capability towards higher SS status.

## 1. Introduction

It is well known that cardiovascular disease is the leading cause of increased morbidity and mortality worldwide. One of the key surgical procedures that patients with coronary artery disease (CAD) are often subjected to is coronary artery bypass grafting (CABG). However, the cardiopulmonary bypass (CPB) pump increases systemic oxidative stress (OS) and often leads to postoperative complications [1, 2]. This is related to ischemia and harmful effects of extracorporeal circulation (ECC) which induce free radical generation production. Additionally, OS becomes more pronounced during reperfusion followed by the end of the cardioplegic arrest [1–3].

Therefore, attempts have been made to avoid the CPB deleterious effects and ischemia-reperfusion injury and to reduce the postoperative complications [4] questioning if off-pump coronary artery bypass grafting (OPCAB) on the beating heart may lead to diminishing the OS [5] and reduce the rate of morbidity and mortality compared with conventional on-pump CABG (i.e., CPB) [6, 7]. However, these data are inconclusive since other studies report the opposite results [8, 9].

Indeed, we have previously shown that patients who underwent CPB exhibited increased OS (i.e., higher levels of lipid hydroperoxides and advanced oxidation protein products), as compared with patients that underwent OPCAB during the postoperative period [10].

The pathophysiological trait of the relationship between OS and CABG has not been clearly elucidated. Namely, previous studies have evaluated different prooxidant and antioxidant parameters and different study intervals before and after the surgical procedures [1, 10–13], but discordant results were shown.

In an attempt to overcome the knowledge gap between CABG procedure and the changes in redox homeostasis system, we aimed to explore a wide spectrum of OS parameters through the predefined study times caused by the two different surgical procedures, i.e., CPB or OPCAB, which might add contribution to make a proper choice of adequate surgical technique.

In addition, to obtain deeper insight into the relationship between vessel wall lesion complexity and redox status, we evaluated OS status parameters in specific study times according to SYNTAX (synergy between percutaneous coronary intervention with taxus and cardiac surgery) score (SS), an established angiographic score for evaluation the extensity and severity of CAD [14].

## 2. Patients and Methods

### 2.1. Patients

The current prospective cohort study was conducted at the Medical Military Academy, Belgrade (Department for Cardiac Surgery), in cooperation with the Faculty of Pharmacy, University of Belgrade (Department for Medical Biochemistry). After approval of the Institutional Ethics Committee (number: 29/II-27), each patient before inclusion in the study provided the written informed consent. Detailed methodology was described elsewhere [10]. In brief, a total of 107 patients were consecutively recruited for CABG. The indications for surgery and patients with stable angina pectoris were selected according to guidelines for CABG Surgery (American College Of Cardiology/American Heart Association Task Force on Practice Guidelines).

The two groups of patients were formed. A total of 47 patients were included in the group where examinees underwent CABG on the beating-heart without using CPB (i.e., off-pump coronary artery bypass grafting-OPCAB), whereas a total of 60 patients encompassed the group undergoing CABG using CPB on the potassium arrested heart (i.e., on-pump coronary artery bypass grafting-CPB).

Patients with metabolic diseases (other than diabetes mellitus), recent myocardial infarction or perioperative myocardial infarction, unstable angina pectoris, heart failure, mediastinal bleeding, massive postoperative and previous stroke or transient ischemic attack, reoperation, chronic renal failure, malignant or autoimmune diseases, acute infections, and use of immunosuppressive drugs and dietary supplements were excluded from this study.

Preoperatively, the SS [14] was calculated for each patient. SS is widely used for prediction events following percutaneous coronary intervention. In line with this, all patients were divided into low (i.e., SS < 22), moderate (i.e., SS between 22 and 30) and high SS subgroup (i.e., SS > 30).

Clinical SYNTAX score (CSS) was also calculated [15]. This score includes age, ejection fraction, and creatinine clearance and should better predict the complexity of patients status than SS alone, which is rather anatomical measure of lesion complexity. By adding CSS calculation, we got better evidence about overall functionality of CAD patients, i.e., some critical functions, like renal function, affected by the main disease.

Two-dimensional transthoracic echocardiography using biplane modified Simpson method was applied as the most commonly used diagnostic procedure to assess left ventricular ejection fraction (LVEF) clinically.

The EuroSCORE II [16] was developed to predict in-hospital mortality after cardiac surgery and includes the following risk factors: dyspnea, angina, extracardiac arteriopathy, poor mobility, previous cardiac surgery, renal dysfunction, active endocarditis, critical preoperative state, LV function or LVEF, urgency of procedure, and recent myocardial infarction.

### 2.2. Anesthesia

A standardized anesthetic technique was applied to all patients which was induced by (1 mg/kg) sufentanyl (0.5–1 *μ*g/kg) and etomidate (0.1–0.2 mg/kg). General anesthesia was maintained with sufentanyl (0.5–1 *μ*g/kg/h) and sevoflurane (0.6–1.0%).

### 2.3. Surgical Procedure

A procedure of midline sternotomy and harvesting of left internal mammary artery as a pedicle and saphenous vein grafts was performed and was followed by full exposure of the coronary artery branches for revascularization.

#### 2.3.1. CPB Group

A procedure of ascending aortic cannulation and two-stage venous cannulation in the right atrium was instituted for CABG using a CPB pump. A low-prime oxygenator with an incorporated cardiotomy reservoir (Sorin Inspire 8, Sorin Group, Mirandola, Italy) and a roller pump (Stockert-S5, Sorin Group, Munich, Germany) were used for CPB circuit.

The perfusion pressure between 50 and 80 mmHg and the nonpulsatile pump flow at 2.2-2.4 L/min/m^2^ were maintained during CPB. The protection of miocardium was maintained with 4°C homemade cold potassium cardioplegia. All distal anastomoses of the bypass grafts were done on the arrested heart.

#### 2.3.2. OPCAB Group

The Octopus® IV Tissue Stabilizer and Starfish™ 2 Heart Positioner (Medtronic, Inc., Minneapolis, MN, USA) was used for the maintenance of mechanical stability of the coronary arteriotomy area.

Soft plastic intraluminally coronary flow-shunt (Medtronic, Clearview®, Medtronic, Inc., Minneapolis, MN, USA) was used for myocardial protection. It was always passed into the coronary arteriotomy to prevent the ischemia of myocardium during placement of distal anastomosis. All of the anastomoses were done on the beating heart.

### 2.4. Sample Collection and Analyses

The venipuncture was performed at 6 time intervals: before surgery (t1), immediately after intervention (t2), 6 h (t3), 24 h (t4), 48 h (t5), and 96 h after termination of the operation (t6).

This procedure was the same for OPCAB and CPB group.

The central venous line from the jugular internal vein was used for such purpose. The K_2_EDTA sample tubes (for plasma) and serum sample tubes were provided. The serum samples were left to clot for 30 minutes and then sera were obtained by centrifugation 3000 rpm for 15 minutes and kept at −80°C, until analysis. The plasma samples were provided after centrifugation of samples in K_2_EDTA tubes 3000 rpm at room temperature for 15 minutes and kept at −80°C, until analysis.

### 2.5. Measurement of Oxidative Stress (OS) Status Parameters

All OS status parameters were determined on an ILAB 650 analyzer (Instrumentation Laboratory, Milan, Italy).

Superoxide anion was measured in plasma following the method of Auclair and Voisin [17], as a rate of nitroblue tetrazolium (NBT) reduction for determination of the rate of superoxide anion generation.

Levels of prooxidant-antioxidant balance (PAB) were determined in serum by the method of Alamdari et al. [18] using 3,3′, 5,5′-tetramethylbenzidine as a chromo gen.

Malondialdehyde (MDA) levels were measured in serum as a thiobarbituric acid reactive substance [19].

Superoxide-dismutase (SOD) activity was determined in plasma according to method of Misra and Fridovich [20] depending on the capability of the SOD to inhibit autooxidation of adrenalin in alkaline medium.

Ischemia-modified albumin (IMA) was measured in serum by method of Bar-Or et al. [21], and values are reported as absorption units (ABSU).

Total antioxidant status (TAS) and total oxidant status (TOS) were determined in serum with o-dianisidine [22] and an ABTS as a chromogen [23], respectively.

Oxidative stress index (OSI) was calculated as previously described: OSI (arbitrary unit) = TOS (*μ*mol H_2_O_2_ equivalent/L)/TAS (*μ*mol Trolox equivalent/L) × 100 [24].

### 2.6. Statistical Analysis

A SPSS statistical package (version 18.0 for Windows, SPSS, Chicago, IL, USA) was used for statistical analysis. The sample size for each group (OPCAB and CAB) was calculated by GPower software, and chosen *α* level and power were 0.05 and 0.80, respectively. We approximated partial eta squared to be small (0.05), so the effect size f was 0.15. In addition, we approximated correlation among repeated measures and nonsphericity correction to be 0.6 and 1, respectively. The calculated sample size was 40 for each group. We increased that number by 15%, considering that nonparametric tests will be used to compare some parameters. The data are presented as mean ± standard deviation (SD), median (interquartile range), or counts and percentages (%). For repeated measured ANOVA, sphericity was tested using Mauchly's test, and Levene's test was used for ANOVA homogeneity of variance checking. ANOVA repeated measures as the nonparametric Friedman test, followed by the Wilcoxon signed-rank test, Kruskal-Wallis nonparametric analysis of variance, followed with the Mann-Whitney *U* test were used for testing the differences between groups. A chi-square test was used for testing the differences between categorical data. A Spearman's correlation analysis with correlation coefficient (*ρ*) was applied to examine the relationships between variables. For estimation of clinical and general factors that could influence SS value a logistic regression model of integrated parameters (t1-t6 study times) was applied. We used logistic regression analysis to determine the predicted probabilities of each model formed from redox parameters measured in different time points for further receiver-operating characteristic (ROC) analysis. Accuracy of each logistic model was calculated for discrimination high from low SS patients. In all analyses, a *p* value <0.05 was regarded to be statistically significant.

## 3. Results

Regarding SYNTAX Score subgroups, patients did not differ by age, body weight, body mass index (BMI), LVEF, and EuroSCORE-Logistic index. They did not also differ in glucose, creatinine, total proteins, eGFR and hematologic parameters ((white blood cell count (WBC), red blood cell count (RBC), hemoglobin (Hgb), and platelet count (PLT)), therapy usage, and percentage of smokers. Patients with higher SS values had significantly higher extracorporal circulation time (ECC) and aortic cross clamp time (ACC), so as the average bypass number (Table 1).

The several main characteristics of CPB procedure such as aortic cross clamp time (ACC) and extracorporal circulation time (ECC) are significantly higher in patients with higher SS, which implies the more complicated artery lesion condition in patients subjected to this procedure. We have also calculated CSS. With SS increase, we have also found significant CSS increase (Table 1).

**Figure 1:**
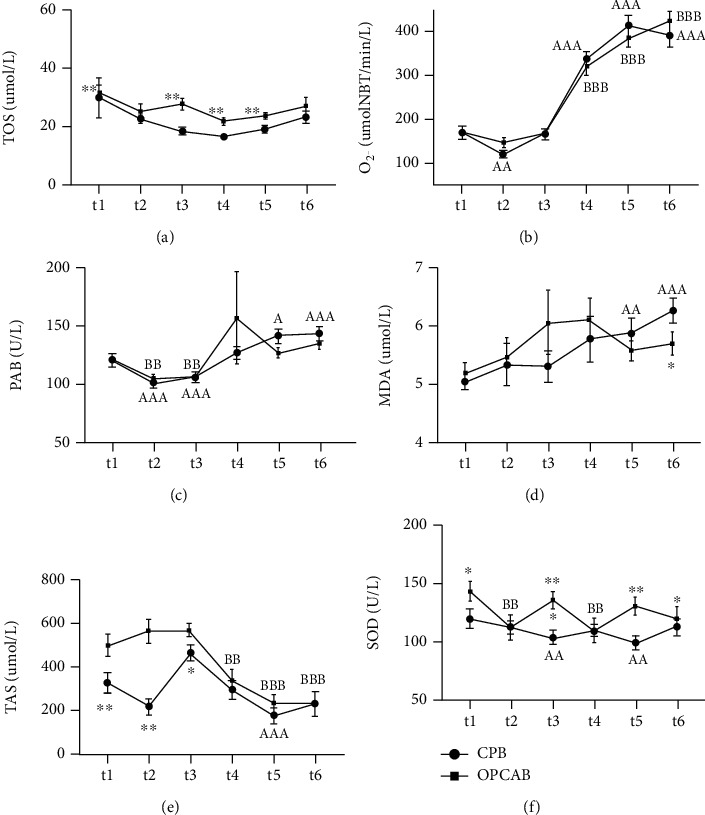
Dynamics of oxidative stress parameters' changes in CABG, precisely two surgery types, i.e., CPB and OPCAB before surgery and after the procedure in defined study points. (a) Total oxidative status (sTOS), (b) superoxide anion (pO_2_^.–^), (c) prooxidative-antioxidative balance (sPAB), (d) malondyaldehide (sMDA), (e) total antioxidative status (sTAS), and (f) superoxide-dismutase (pSOD). ^∗^, ^∗∗^, ^∗∗∗^*P* < 0.05; 0.01; 0.001 OPCAB vs. the same time point in CPB group. ^a, aa, aaa^*P* < 0.05; 0.01; 0.001 in CPB group (t2-t5) vs. baseline. ^b, bb, bbb^*P* < 0.05; 0.01; 0.001 in OPCAB group (t2-t5) vs. baseline. s: serum; p: plasma.

The OS status parameters change through the predefined study times according to implemented surgical technique (i.e., CPB or OPCAB) are presented in Figure 1.

TOS decreased in both study groups, compared to baseline values, but this decrease did not reach statistical significance. TOS values were higher in OPCAB compared to CPB group in several study points.

Superoxide anion showed significant increase in t3-t6 study times, in CPB, so as in OPCAB group, with comparable values in both study groups, and the highest values at t6 study point. Similar way of increase has also seen for PAB parameter. MDA significantly increased only in CPB group in t5 and t6 interval. MDA was significantly higher in CPB group compared to OPCAB in t6 study point.

CPB patients had significantly lower TAS compared to OPCAB patients at the beginning and also in t2 and t3 study points. In CPB, we noticed the initial decrease in TAS values, then sharp increase and decrease till the end of the study period. In OPCAB, we found initial increase in TAS and from the t3 (24 h after the surgery end), significant decrease compared to baseline value. CPB patients had significantly lower SOD activities compared to OPCAB, baseline, and in several study points, when SOD increased in OPCAB patients.

CPB patients had higher IMA baseline than OPCAB group. Initially, IMA rose in CPB, in t2 point, and after that we could not report any significant change in this parameter till the study end.

Parameters in specific study times, which showed significant difference according to SS level (i.e., low, intermediate, and high SS subgroups) are presented at the Table 2.

It is clear that TAS level is significantly lower in patients with high SS level, baseline so as in the first two study times (till 24 h). Also, SOD decrease along with SS increase was obvious, so as TAS/TOS ratio, which is also representative of antioxidative potential. Unexpectedly, TOS was also lower in high SS group of patients. PAB was significantly higher in patients with high SS compared to low and moderate groups. OSI index (TOS/TAS ratio) was also higher in high SS group than in low and moderate groups.

Correlation analysis of OS status parameters and SS value showed that TAS and TOS parameters had significant negative correlation with SS level for the most study point times.

SOD also showed significant inverse correlation with SS level. PAB 48 h after the surgery showed positive correlation with SS level. Both results are expected having in mind OS parameters nature (Table 3).

Correlation in subgroups according to SS level showed the following: significant negative correlation between IMA and SS in Low SS group, positive TAS correlation in moderate SS group and negative correlation in high SS group, and positive MDA correlation with SS level in high SS group (Table 4).

**Figure 2:**
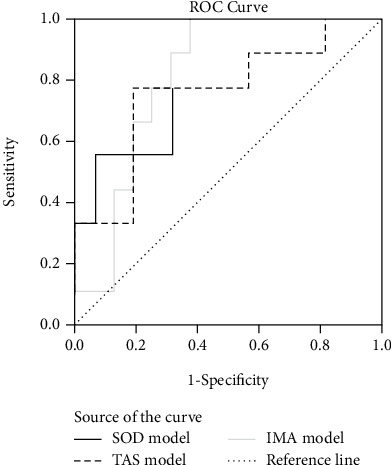
ROC analysis of different OSS parameters as SYNTAX Score value predictors. AUC (SE): area under the curve (standard error); 95^th^ CI (confidence interval); *P*: significance. Logistic regression models of integrated parameters (t1-t6 study times) are presented.

ROC analysis was performed to find the most significant predictors of SS level (low vs. high SS subgroups). Using logistic regression analysis for integrated OS status parameter models generation, the best SS level predictors were SOD activity, IMA, and TAS level (Figure 2).

Logistic regression analysis showed that SOD and IMA had very good discriminatory capability (AUC = 0.840 and AUC = 0.813, respectively) [25].

Out of 107 CABG patients included in the current study, a total of 3 postoperative complications were noted in the OPCAB group, and 12 in the CPB group and the most prominent ones were leg wound infection, pleural effusion, pulmonary thromboembolism, and shallow sternal wound infection.

## 4. Discussion

To our knowledge, this is the first study that examined the broad spectrum of OS biomarkers in patients undergoing CABG in relation to different surgery modalities. Several important findings of the current study need to be acknowledged. According to the main aim of this project, we have followed OS status parameters (i.e., IMA, MDA, superoxide anion, PAB, TOS, TAS, and SOD) change through the predefined study times to obtain deeper knowledge into the redox homeostasis before and after CABG intervention. We have also analyzed the difference in OS generation caused by the two different surgical procedures, i.e., CPB or OPCAB and found that CPB patients were in more severe ischemia (as measured with IMA) baseline than OPCAB group. Furthermore, IMA rose in CPB patients immediately after the surgery end and after that no significant change in this parameter was observed.

Following the change of OS status parameters before and after the surgical intervention in CPB and OPCAB group, we have observed the decrease (but without statistical significance) in TOS in both study groups, compared to the levels before the operation. We have shown that TOS levels, as a measure of peroxide generation [23], were lower in CPB compared to OPCAB group in several study points. It seems that dynamics of this parameter change was not compatible with our study times. Precisely, peroxides generation is probably the fast process, and this kind of reactive species are subjected to rapid decomposition, during the surgical procedure, i.e., the times which we did not include in this study.

After the initial decrease, superoxide anion showed significant increase in other study times in both, OPCAB and CPB group, exhibiting the highest levels at the end of the observed period. Similar way of increase was also seen for PAB. MDA significantly increased only in CPB group towards the end of the observed period. MDA is a marker of late peroxidation phase [19], and we have shown that it was significantly higher in CPB group compared to OPCAB in 96^th^ h after the surgery completion.

CPB patients had significantly lower TAS levels compared to OPCAB patients at the beginning and also 6 h after intervention. CPB patients had significantly lower SOD activities compared to OPCAB, baseline, and in several study points, when SOD increased in OPCAB patients. SOD dynamics was interesting and could be analyzed with the changes of other oxidative status factors, primarily with superoxide anion dynamics, which is a substrate for SOD enzymatic activity [20].

Luyten et al. [26] found an increase in SOD activity, which returned to the baseline values 24 h after the surgical procedure. At the same time in the mentioned study, the authors reported huge increase of the total antioxidant capacity of 60%. In our current study, antioxidants (enzymatic and nonenzymatic) showed also initial increase and then decrease till the study end (96 h).

In our previous study, we have recorded higher levels of lipid hydroperoxides and advanced oxidation protein products in the CPB, as compared with OPCAB group during the postoperative period, showing that OS was higher in the CPB group which could be attributed to more harmful conditions of the CPB patients or their diminished ability to respond to the OS threat [10].

**Figure 3:**
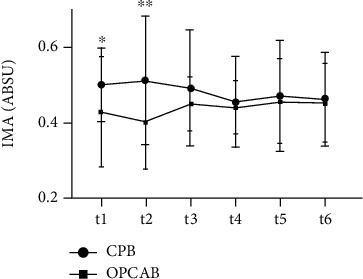
Ischemia-modified albumin (sIMA) concentration in CABG subjects and according the two surgery types (CPB and OPCAB) before surgery (t1) and after the procedure (t2-t6). ^∗^, ^∗∗^, *P* < 0.05; 0.01 OPCAB vs. the same time point in CPB group; s: serum.

By measuring IMA, we aimed to follow up changes in ischemic condition in CAD patients. Initially, IMA has increased after the surgery in our group of patients, but earlier in CPB compared to OPCAB patients (Figure 3). Kanko et al. [13] also found significantly increased IMA values in a group of CPB patients. Taking into account that IMA is a marker of the early myocardial ischemia, we assumed that IMA measurement is worthwhile in cardiovascular bypass surgery settings. Thus, IMA measurement could contribute to the earlier treatment and better follow-up of the CABG patients. According to Bar-Or et al. [21], a value of 0.400 ABSU represents the cut-off point which delineates ischemic condition. It was obvious that our patients' values were far above 0.400 ABSU in the whole course of the observed period, suggesting that examined CAD patients were in chronic ischemia state, which was more pronounced in CPB compared to OPCAB patients. Ischemia may lead to increased production of reactive oxygen species and consequent modification of albumin. These processes result in an increased IMA formation, which was shown to be a reliable marker in detection of the early stages of ischemia, before the occurrence of necrosis [13].

In order to get better insight into the relationship between vessel wall lesion complexity and redox status, we have divided study patients at low, moderate, and high SS subgroups and compared OS status parameters in specific study times. Namely, it was previously shown that patients with SS above 22 (boundary between low and moderate atherosclerotic lesion) according to the results of Syntax score study [14] should be subjected exclusively to CABG surgery in order to avoid adverse cardiovascular events and need for revascularization. The several main characteristics of CPB procedure, such as aortic cross clamp time (ACC) and extracorporal circulation time (ECC), were significantly higher in patients with higher SS, which implies the more complicated artery lesion condition in patients subjected to this procedure. With SS increase, we have also found significant CSS increase, which suggests general worsening of patients' systemic condition as a consequence of advanced atherosclerosis (Table 1).

Additionally, we revealed that the antioxidative potential (i.e., TAS, SOD, and TAS/TOS ratio) was significantly lower, whereas parameters that show OS balance (i.e., PAB and OSI index) were significantly higher in patients with high SS compared to low and intermediate SS group. Unexpectedly, TOS was also lower in high SS group of patients, and this finding should be subjected to further analysis.

Thereafter, ROC analysis was performed to find the most significant predictors of SS level (low vs. high SS subgroups). Using logistic regression analysis for OS status parameter generation, the best SS level predictors were SOD activity, IMA, and TAS level. SOD and IMA showed very good discriminatory capability towards higher SS status.

Antioxidant capacity has previously been recorded as a predictive marker in determining postoperative complications [12].

The CPB technique is a main reason for ischemia-reperfusion which causes the release of superoxide anion, primarily by the xanthine oxidase (XO) system [27].

Products of purine bases (hypoxanthine and xanthine) accumulate in ischemic tissues, and XO was suggested to be a key source of reactive oxygen species (ROS) during ischemia/reperfusion. With the onset of ischemic state, the accumulation of hypoxanthine produced via the catabolism of ATP, and a consequent conversion of xanthine dehydrogenase (XDH) to XO occurs, which is the key source of ROS [28]. Along with the reperfusion process, it is assumed that oxygen and accumulated hypoxanthine react with XO leading to increased production of superoxide anion [27].

In addition, treatments with allopurinol and SOD were shown to be efficient in attenuating the response of vascular permeability induced by ischemia/reperfusion process, thus suggesting that XO-derived superoxide, a substrate for SOD enzymatic activity [20], was involved in the endothelial dysfunction [27].

Despite the fact that we have included a wide spectrum of parameters of redox homeostasis in this study, we should mention its several drawbacks. The limitation of the current study lies in the fact that it included a relatively small number of patients, and all measurements of oxidative stress parameters were performed once. However, this is the standard procedure for determination of oxidative stress parameters because all methods are validated, the calibration was performed, and the reliability of results and the quality control are maintained by measuring normal and abnormal control material before analyses.

We were also limited for determination of lipid metabolism parameters which may affect the results of the study [29, 30]. Moreover, we were not able to exclude type 2 diabetes, smoking, and medications other than immunosuppressive drugs and dietary supplements, which all might affect the results of the study. In addition, a control group of healthy subjects could have been useful to compare the baseline oxidative stress status parameters with the study group. Also, this is a single-center study. Therefore, new multicenter studies with larger sample size and with control group included would be of benefit to better evaluate the role of oxidative stress in the mentioned pathology and to validate our results.

## 5. Conclusion

The results of the current study revealed that patients with high SS exhibited pronounced redox imbalance reflected by increased prooxidant species, while reduced antioxidant elements compared to patients with low and intermediate SS. Additionally, two redox status opposite features, both with protein nature, i.e., one important enzymatic antioxidant (SOD) and the other product of oxidative stress burden (IMA), could predict CAD severity and complexity, determined as SS values. This study adds information about more severe ischemic condition of CPB patient baseline compared with OPCAB group, and also, we got insight into severe ischemia development in the CPB patients after the surgery. Importantly, this ischemia did not persist in subsequent study points, which is encouraging having in mind worse condition of these patients compared to OPCAB ones.

Taking all these into account, further investigation of redox status parameters would be of great importance in different cardiac surgery modalities which might enable to reduce the deleterious effects of these procedures, by inclusion of some preoperative and/or postoperative antioxidants as supportive therapy.

## Figures and Tables

**Table 1:** General clinical and surgery related parameters according to SYNTAX Score tertile values.

Parameter	Low SS < 22 (15.6 ± 4.8)	Moderate SS 22-32 (27.1 ± 3.2)	High SS > 32 (38.6 ± 6.1)	*P*
*n*	34	39	33	/
Age (years)	63.2 ± 9.6	62.2 ± 9.4	67 ± 9.1	ns
Body weight (kg)	81.1 ± 12.7	81.1 ± 10.2	84.4 ± 14.5	ns
BMI (kg/m^2^)	27.7 ± 3.1	27.6 ± 3.4	28.8 ± 4.4	ns
sGlucose (mmol/L)	6.40 ± 2.02	6.34 ± 2.12	6.77 ± 2.55	ns
sCreatinine (*μ*mol/L)	85.1 ± 25.8	95.0 ± 58.1	89.5 ± 27.7	ns
sTotal proteins (g/L)	71.2 ± 5.1	71.0 ± 5.4	70.8 ± 4.8	ns
eGFR (mL/min/1.73 m^2^)	83.6 ± 21.4	83.0 ± 26.1	79.1 ± 20.1	ns
WBC (×10^9^/L)	7.0 ± 1.8	6.9 ± 1.6	7.1 ± 1.8	ns
RBC (×10^12^/L)	4.6 ± 0.5	4.6 ± 0.5	4.6 ± 0.6	ns
Hgb (g/L)	134 ± 14	136 ± 14	135 ± 12	ns
PLT (×10^9^/L)	230 ± 60	233 ± 52	220 ± 69	ns
LVEF (%)	52.0 ± 8.7	50.0 ± 9.9	47.6 ± 10.9	ns
EuroSCORE-logistic	5 (3-8.25)	5 (3-7)	7 (5-10)	ns
Clinical SS	19.1 (13.3-29.1)	33.6 (26.6-43.8)^aaa^	50.4 (43.8-81.2)^aaa,bbb^	<0.001
ECC (min)	0 (0-0)	72 (0-87)^aa^	81 (72.8-114.5)^aaa,bb^	<0.001
ACC (min)	0 (0-0)	29 (0.0-49)^aa^	44 (36-56)^aaa,bb^	<0.001
Bypass number	2.06 ± 0.49	2.33 ± 0.54	2.59 ± 0.64^a^	<0.05
*β*-Blockers, *n* (%)	30 (88.2%)	38 (97.4%)	33 (100.0%)	0.055
ACE inhibitors, *n* (%)	27 (79.4%)	37 (94.9%)	29 (87.9%)	ns
Calcium-antagonists, *n* (%)	11 (32.4%)	13 (33.3%)	12 (36.4%)	ns
Nitrates, *n* (%)	29 (85.3%)	34 (87.2%)	32 (97.0%)	ns
Statins, *n* (%)	22 (64.7%)	30 (76.9%)	24 (72.7%)	ns
Oral antidiabetics, *n* (%)	8 (23.5%)	5 (12.8%)	8 (24.2%)	ns
Diuretics, *n* (%)	10 (29.4%)	9 (23.1%)	16 (48.5%)	ns
Smokers, *n* (%)	23 (67.6%)	30 (76.9%)	22 (66.7%)	ns

BMI: body mass index; LVEF: left ventricular ejection fraction; ECC: extracorporal circulation time; ACC: aortic cross clamp time; *P*: ANOVA or Kruskall-Wallis nonparametric analysis of variance; Tuckey-Snedecor post hoc test. ^a,aa,aaa^: *P* < 0.05, 0.01, 0.001 vs. low SS group; ^bb^*P* < 0.01 vs. moderate SS group.

**Table 2:** Oxidative stress status parameters according to SYNTAX Score tertile values in different study times depending on significant change through SS subgroups.

Parameter	Low SS < 22 (15.6 ± 4.8)	Moderate SS 22-32 (27.1 ± 3.2)	High SS > 32 (38.6 ± 6.1)	*P*
sTAS t1 (*μ*mol/L)	497 (331-564)	393 (157-689)	247 (113-370)^aa^	<0.05
sTAS t2 (*μ*mol/L)	476 (261-653)	238 (206-575)	166 (2.00-290)^aaa,bb^	<0.01
sTAS t3 (*μ*mol/L)	568 (438-638)	617 (434-727)	357 (220-526)^aa,bb^	<0.01
sTOS t3 (*μ*mol/L)	26.4 (20.5-32.7)	18.3 (15.6-22.9)^a^	15.1 (12.5-23.0)^aa,b^	<0.05
sTOS t4 (*μ*mol/L)	20.8 (17.4-24.9)	17.7 (14.3-20.5)	16.7 (10.8-22.6)^a^	<0.05
pSOD t5 (U/L)	144 (120-156)	121 (60-135)^a^	111 (76-122)^aa^	<0.05
sPAB t5 (U/L)	125 (115-145)	118 (107-136)	145 (125-179)^a,b^	<0.05
TAS/TOS t2	20.3 (9.5-28.0)	14.0 (7.8-30.6)	7.3 (0.1-13.7)^aa,bb^	<0.05
OSI index t2	0.05 (0.04-0.11)	0.07 (0.03-0.13)	0.14 (0.07-7.87)^aa,bb^	<0.01

*P*: Kruskall-Wallis test, ^a,aa,aaa^*P* < 0.05; 0.01; 0.001, respectively, vs. low SS group (<22 points), Mann-Whitney *U* test; ^b,bb,^*P* < 0.05; 0.01, respectively, vs. moderate SS (22-32 points), Mann-Whitney *U* test; s: serum; p: plasma.

**Table 3:** Spearman's nonparametric correlation between SYNTAX Score value and OS status parameters.

OSS parameter	SYNTAX score
sTAS, *μ*mol/L t1	-0.318^∗^
sTAS, *μ*mol/L t2	-0.417^∗∗∗^
sTAS, *μ*mol/L t3	-0.249^∗^
sTAS, *μ*mol/L t6	-0.259^∗^
sTOS, *μ*mol/L t1	-0.259^∗^
sTOS, *μ*mol/L t3	-0.418^∗∗^
sTOS, *μ*mol/L t4	-0.331^∗∗^
sTOS, *μ*mol/L t5	-0.298^∗^
sTOS, *μ*mol/L t6	-0.247^∗^
pSOD, U/L t3	-0.310^∗^
pSOD, U/L t5	-0.368^∗∗^
sPAB, U/L t5	0.256^∗^

Data were shown with (*ρ*)-Spearman's correlation coefficient. ^∗^,^∗∗^*P* < 0.05, 0.01, respectively, are only for the significant correlation between SS and OS status parameters in different study times; s: serum; p: plasma.

**Table 4:** Spearman's nonparametric correlation in SYNTAX Score subgroups (SS < 22, SS 22-32, SS > 32).

Parameter, study time	Low SS < 22	Moderate SS (22-32)	High SS > 32
sIMA, ABSU t4	-0.460^∗^	/	/
sIMA, ABSU t5	-0.455^∗^	/	/
sTAS, *μ*mol/L t3	/	0.474^∗^	/
sTAS, *μ*mol/L t4	/	/	-0.526^∗^
sMDA, *μ*mol/L t1	/	/	0.551^∗^

Data were shown with (*ρ*)-Spearman's correlation coefficient. ^∗^*P* < 0.05 for the significant correlation between SS and OS status parameters in SS level subgroups. s: serum.

## Data Availability

The data will be available upon reasonable request (contact person: aleksandranklisic@gmail.com).
